# Plaque Length Predicts the Incidence of Microembolic Signals in Acute Anterior Circulation Stroke

**DOI:** 10.1155/2021/2005369

**Published:** 2021-07-28

**Authors:** Liming Zhao, Hongqin Zhao, Yicheng Xu, Aijuan Zhang, Jiatang Zhang, Chenglin Tian

**Affiliations:** ^1^Department of Neurology, Medical School of Chinese People's Liberation Army, Chinese People's Liberation Army General Hospital, Beijing 100039, China; ^2^Department of Neurology, Affiliated Hospital of Weifang Medical College, Weifang 261055, China; ^3^Department of Neurology, Affiliated Hospital of Qingdao University, Qingdao 266000, China; ^4^Department of Neurology, Aerospace Center Hospital, Beijing 100049, China; ^5^Department of Neurology, Weifang People's Hospital, Weifang 261021, China

## Abstract

Microembolic signals (MES) of the carotid artery are associated with plaque destabilization and reoccurrence of stroke. Previous studies have focused primarily on the degree of carotid artery stenosis and plaque components, and the relationship between plaque length and microembolic sign has received little attention. We aimed to find the association between carotid plaque length (CPL) and the presence of MES. We conducted a retrospective observational cross-sectional study. A total of 84 acute anterior-circulation ischemic stroke/transient ischemic attack (TIA) patients with carotid artery atherosclerosis were classified into an MES-positive (MES+) group and MES-negative (MES−) group. We measured multiple parameters of carotid plaque size (length, thickness) in each patient and evaluated the relationship between different plaque parameters and occurrence of MES. We found that male, carotid artery stenosis (CAS), CPL, carotid plaque thickness (CPT), and intima-media thickness (IMT) of the carotid artery were each significantly different between two groups (all *P* < 0.05). The multivariate analysis showed CPL (odds ratio (OR), 1.109; 95% CI, 1.044–1.177; *P* = 0.001) to be independently associated with the presence of MES. The areas under the ROC curves (AUCs) for CPL for predicting MES were 0.777 (95% CI, 0.640–0.914; *P* < 0.001). The cutoff value of CPL for predicting MES was 16.7 mm, with a sensitivity of 88.2% and a specificity of 77.6%. We found that CPL was a meaningful independent predictor of MES. Therefore, CPL may be useful for risk stratification of long and nonstenotic plaques in anterior circulation stroke.

## 1. Introduction

Microembolic signals (MES) of the cerebral artery are associated with plaque destabilization and predict the occurrence of stroke [1–5]. Furthermore, MES can cause recessionary cognition [6]. Detection of MES may provide a diagnostic stratification in patients with asymptomatic carotid stenosis and aid in optimizing therapies for such patients [1], as well as serve as a tool for elucidating the mechanisms of stroke and evaluating efficacies of antiplatelet therapies [2, 5, 7]. Hence, MES should be widely used in observational and interventional studies [7, 8].

A previous study [9] found that carotid plaque thickness (CPT) > 3 mm may be a source of thromboembolic stroke. Another study shows CPT > 3 mm failed to be significantly different with ipsilateral embolic stroke of undetermined source [10]. Growth of plaque length of carotid artery is faster than their corresponding thicknesses [11]. Thus, plaque length may be an underestimated indicator of carotid artery atherosclerosis. Only a few studies focus on the search of CPL [12, 13]. Therefore, the relationship between lengths of plaques of the carotid artery and MES requires further investigation. Importantly, assessment of the lengths of carotid plaques may be useful for discerning high-risk plaques. The purpose of our study was to discover the relationship between CPL and MES.

## 2. Materials and Methods

### 2.1. Patients and Study Design

This was a retrospective observational cross-sectional study. We investigated the relationship between CPL and MES lasting 60 min during Transcranial Doppler (TCD) monitoring within 72 h after the onset of acute stroke. Consecutive patients with acute anterior-circulation ischemic stroke or transient ischemic attack (TIA), admitted to the Department of Neurology at Weifang Brain Hospital, were enrolled in the present study from January 2015 through October 2019. Stroke was diagnosed based on imaging characteristics obtained via magnetic resonance imaging (MRI) and neurological deficits lasting for more than 24 h. TIA was defined based on the criteria of the American Heart Association/American Stroke Association (AHA/ASA) [14]. CAS was diagnosed based on ultrasound examination. Our study was approved by Changyi People's Hospital Ethics Committee. The approval no. of the Ethics Committee was CYRM20171014. Our study was a retrospective observational study. Patient informed consent for inclusion in this study was waived. The patients' general data, relevant medical history, treatments, and laboratory examinations were evaluated and recorded by a neurologist.

Exclusion criteria for candidate patients were as follows: (1) <40 years old, (2) carotid artery occlusion or middle cerebral artery occlusion, (3) the absence of a temporal acoustic window for TCD monitoring, (4) bilateral anterior infarctions and/or anterior- and posterior-circulation infarctions, (5) cardioembolic stroke or strokes with other etiologies, (6) severe nephritis or liver disease or definitive or suspected cancer, (7) no enduring MES for 60 min during TCD monitoring, and (8) a history of carotid endarterectomy or a carotid artery stent.

### 2.2. Assessment of CAS via Ultrasonography

Ultrasound examination was conducted by two skilled doctors. The carotid artery stenosis (CAS) was defined by criteria of ECST (arrange 50% to 99%) (16). Other ultrasonic parameters [15] that we assessed were as follows: (1) CPL was defined as the maximum length of all ipsilateral carotid artery plaques [13]; (2) CPT was defined as the maximal thickness of all plaques within ipsilateral carotid arteries; (3) resistance index (RI) was defined by the Mannheim Carotid IMT Consensus, and IMT was assessed at the thickness of segments without plaques and was measured in the far wall of the common carotid artery at approximately 10 mm proximal to the carotid artery bifurcation, as previously described [16]; (4) plaques of ipsilateral carotid arteries were categorized as either predominantly echolucent, predominantly echogenic, or as mixed echolucent/echogenic; and (5) ulcerative plaques were defined by common criteria in ultrasonography [17], including plaque surface craters measuring 2 × 2 mm or those with concavity with an echogenic line at the plaque base. Timing of ultrasound examination was not limited, and most of the patients were detected within 3 days after stroke.

### 2.3. Assessment of MES via TCD Monitoring

MES were detected via TCD monitoring (Delica EMS-9A); for this purpose, skilled technicians fixed a 2 MHz probe to the patients head frame and monitored occurrence of MES in the initial and distal segments of the symptomatic middle cerebral artery for 1 hour. All patients were detected within 3 days after stroke/TIA. Distances ≥ 6 mm between two points were applicable for MES monitoring. Typically, the middle cerebral artery was monitored at depths between 50 and 65 mm. The sample volume based on a nearly 8 mm vessel length, combined with a relative low gain, was used to distinguish emboli signals from background noise. The skilled technicians identified the presence of MES as unidirectional, short duration signals (less than 0.3 s) with intensity threshold above 3 dB, accompanied by “chirping” sound and occurring randomly throughout the cardiac cycle, as previously described [18].

### 2.4. Statistical Analysis

The SPSS 22.0 software package (Chicago, IL, USA) was utilized for data analysis. Quantitative data are expressed as the mean ± standard deviation, while qualitative data are expressed as frequencies and percentages. After testing for normality, quantitative data were compared between two groups by *t*-tests, and qualitative or categorical data were compared by *χ*^2^ tests or Fisher's exact texts.

Statistically significant factors (*P* < 0.05) in univariate analyses were entered into stepwise forward logistic regression analysis to identify the independent factors for MES. Odds ratios (ORs) and their 95% CIs were used to evaluate the independent contributions of significant factors. The Hosmer-Lemeshow test was used to estimate the appropriateness of the model.

We measured the correlations between CPT and CPL by calculating Pearson correlation coefficients.

Receiver operating characteristic (ROC) curves were obtained to determine the optimal cutoff values for the independent risk factors, as well as their sensitivities, specificities, and areas under the ROC curves (AUCs). *P* < 0.05 was reckoned statistically significant.

## 3. Results

### 3.1. Baseline Demographics

**Figure 1:**
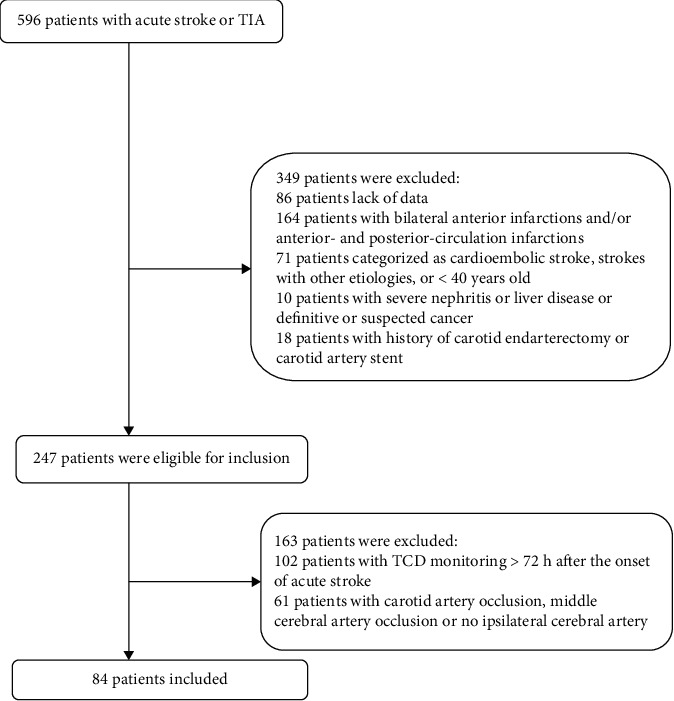
Flowchart of the patients included in the present study.

During the study period, 596 consecutive patients with acute stroke/TIA were potentially eligible for our study. After removing patients that fit the exclusion criteria, a total of 84 anterior-circulation ischemic stroke/TIA patients were enrolled in our present study (Figure 1). These patients' demographic and clinical features are presented in Table 1. The mean age was 62.05 ± 9.29 years. There were 55 (65.5%) males. MES occurred in 17 out of 84 cases. There were no significant differences between MES+ and MES− patients in terms of age or the presence of hypertension, diabetes mellitus, ischemic heart disease, smoking, or drinking. However, the percentage of males in the MES+ group was significantly higher than that in the MES− group. Finally, laboratory parameters—including total cholesterol (TC), triglycerides (TG), low-density lipoprotein (LDL), high-density lipoprotein (HDL), creatinine (Cr), and blood urea nitrogen (BUN) levels—were not significantly different between the MES+ group and MES− group (*P* > 0.05).

### 3.2. Characterization of Carotid Plaques

The patients' ultrasound characteristics are listed in Table 2. CAS, CPL, CPT, and IMT of the carotid artery were each significantly different between the MES+ group and the MES− group (all *P* < 0.05). In contrast, there was no significant difference in the RI between the MES+ group and the MES− group (*P* = 0.707).

We found that the percentage of plaque echolucency was not significantly different between the MES+ group and the MES− group (47.4% vs. 40.8%, respectively).

### 3.3. Multiple Collinear Analysis of Independent Variables and Multivariable Analysis

Variance inflation factors (VIF) of CAS, CPL, CPT, and IMT were less than 5. Multicollinearity was considered nonexistent.

Gender was adjusted because there was a statistically significant difference between male and female. CAS, CPL, CPT, and IMT were entered into logistic regression analysis. Multivariable analysis showed that CPL (OR: 1.109; 95% CI: 1.044–1.177; *P* = 0.001; *B*: 0.103; S.E.: 0.031) was an independent risk factor for MES. Factors including gender, CAS, CPT, and IMT were not in the equation. The *P* value of the Hosmer-Lemeshow test was 0.213.

### 3.4. Correlational Analysis between CPL and CPT

The correlation between CPL and CPT was *R*^2^ = 0.539 and *P* < 0.01, and this correlation was moderate level.

### 3.5. AUC for CPL for Predicting MES

**Figure 2:**
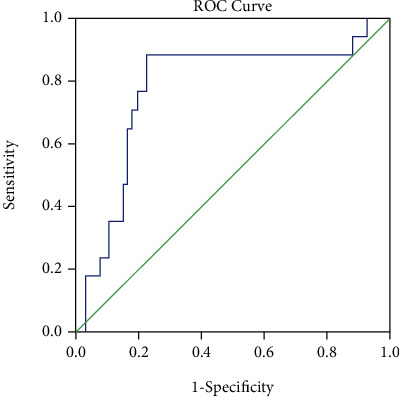
Points along the diagonal dotted line represent an AUC of 0.5. The AUCs for CPL of carotid arteries for predicting MES were 0.777 (95% CI, 0.640–0.914; *P* < 0.001). AUCs: areas under the ROC curves; CPL: carotid plaque length; MES: microembolic signals.

The AUC for CPL for predicting MES was 0.777 (95% CI, 0.640–0.914; *P* < 0.001) (Figure 2). The optimal cutoff value of CPL for predicting MES was 16.7 mm, with a sensitivity of 88.2%, specificity of 77.6%, positive predictive value of 88.2%, and negative predictive value of 77.6%.

## 4. Discussion

The present study evaluated the predictive value of CPL for MES in patients with acute anterior-circulation ischemic stroke/TIA. Vulnerable plaque characteristics such as intraplaque hemorrhages, plaque ulcerations, and thinned or disrupted fibrous caps can be clearly identified by high-resolution magnetic resonance imaging (MRI) or PET-CT [19]. However, due to their relatively low occurrences, costliness, and difficulty to quantify, these specific plaque characteristics have not been widely adopted clinically and may be far from ideal markers of MES [19]. Alternatively, CPL is noninvasive, cost-effective, and easily quantified and may thus be sufficient for predicting MES [13].

In the current study, conventional stroke-related risks such as hypertension, diabetes mellitus, coronary artery disease (CAD), smoking history, and drinking history were not significantly different between the MES+ group and the MES− group. These findings are consistent with those of a previous study [4, 20, 21]. We found that the percentage of males in the MES+ group was significantly higher than that in the MES− group. Related to this finding, a previous study has noted that males have a higher smoking rate, drinking rate, and other increased risk factors compared to those of females in China [18]. Our study and previous studies all found that there were no significant differences about plasma lipid level (TC, TG, LDL, or HDL), Cr, and BUN between the two groups [20]. Previous studies also deem that dual antiplatelet therapy can reduce MES more effectively than single antiplatelet therapy [2, 4]. In our study, dual antiplatelet therapy in the MES− group was nearly two times more frequent than in the MES+ group (35.8% vs. 17.6%, respectively) despite the lack of any statistically significant difference, which may be due to the limited sample size.

In the present study, CAS, CPL, CPT, and IMT were significantly different between the MES+ group and the MES− group. Most of the studies suggested that MES was associated with symptomatic carotid stenosis [5, 22]. However, some studies showed MES had no relationship with symptomatic CAS [23]. IMT is traditionally considered an important arteriosclerosis factor for stroke and its recurrence [21]. Our present study found that IMT was also associated with MES. Only three cases with ulcerative plaques were found in our data, due to rigorous criteria of ulcerative plaques requiring a concavity greater than 2 × 2 mm. In a Chinese study, the proportion of ulcerative plaque was only 8% in 287 patients with moderate internal CAS [24]. Another carotid plaque morphology research also deems the prevalence of ulcerative was fairly low [23], just like our research. These findings suggest that ulcerative plaques (greater than 2 × 2 mm) may not represent a sensitive parameter for predicting MES. Echolucent plaques have been commonly suggested responsible for the presence of MES. Our research showed that the percentage of echolucent plaques was not different between the two groups. Part of the reason may be that plaque morphology will change after stroke or releasing MES.

Logistic regression analysis supported that CPL was independently associated with the occurrence of MES, while CAS, CPT, and IMT were not independent factors for MES in acute anterior stroke. Part of the reason may be due to the limited sample size. The cutoff value of CPL for predicting MES was 16.7 mm. Carotid artery plaques increase in length much faster than in thickness and have a large dynamic scope [11]. In conclusion, CPL may be a meaningful independent sensitive predictor for MES. The relationship between CPL and MES has rarely been reported previously. A recent article reported that plasma osteoprotegerin levels (an inflammatory biomarker) was predictive of MES [20] and that the corresponding AUC (0.734) was also effective; however, laboratory testing for osteoprotegerin levels is not exactly practical compared with CPL in most Chinese hospitals. CPL belongs to plaque morphology parameter and osteoprotegerin level reflects unstable plaque inflammation. Another recent study [13] reported that CPL is an independent indicator of the severity of CAD, and our study showed that CPL would be a useful tool to evaluate high-risk recurrence of stroke instead of CPT and IMT. There were only a few literatures on carotid plaque length. To our knowledge, our study is the first to discover the relationship between CPL and MES. Our research showed that CPL has a correlation with CPT. This relationship needs future exploration.

CPL which is convenient to measure by ultrasound could make up for the limitation of MES. TCD monitoring requires special equipment as well as skilled technicians, and its clinical applications have been limited. Some older people have unilateral or bilateral poor temporal windows and are unable to endure a 1-hour MES monitoring session [20].

CPL may be beneficial for rethinking etiological classifications of stroke via large and nonstenotic plaques. It has been increasingly recognized that there are limitations in Trial of Org10172 in Acute Stroke Treatment (TOAST) classifications, especially in Asian countries with higher rates of LAA (Large Artery Atherosclerosis) [25–28]. For example, the percentage of “undermined stroke” via TOAST classifications is much higher than that of other etiologic stroke classifications [26, 29]. For instance, large and nonstenotic plaques are classified as “undermined stroke” or small-vessel-disease subgroups via TOAST classifications. The total plaque area (TPA ≥ 1.19 mm^2^) is a criterion for indicating nonstenotic LAA stroke with a heavy plaque burden in SPARKLE [30]. Nevertheless, measurement of total plaque area (TPA) is also time-consuming and consequently difficult to be used widely in clinical practice compared with CPL. Our study showed that CPL was a valuable independent marker for the presence of MES, and this result means measuring of CPL would identify the nonstenotic carotid with high risk.

The present study had some limitations. First, the sample sizes were relatively small. Because of our limited sample sizes, a few traditional risk factors failed to meet the criteria for independent risk factors of binary regressions. Therefore, future studies with larger sample sizes and other measurement instruments are needed to confirm or refute our present findings. Second, the MES monitoring that we employed had limitations. Since we only conducted MES monitoring for 1 h, this monitoring time may not have been long enough and may have led to false-negative errors. Finally, since this was a retrospective cross-sectional study, our findings need to be validated by prospective cohort studies in the future.

## 5. Conclusions

Our present findings suggest that ultrasound CPL was a dependent parameter and meaningful predictor for MES, thus suggesting that high CPL may discern the high-risk plaque undermined stroke and small-vessel-disease with high recurrence. CPL may be widely implemented in clinical practice and research.

## Figures and Tables

**Table 1:** Baseline demographics.

	MES+ (*n* = 17)	MES− (*n* = 67)	*T*/*χ*^2^	*P*	
Age (years)	62.53 ± 4.75	61.93 ± 10.15	0.357	0.722	
Gender (male/female)	15 (86.7%)	40 (59.7%)	4.884	0.027	
Hypertension	13 (76.5%)	56 (83.6%)	0.108	0.742	
Diabetes mellitus	6 (35.3%)	11 (16.4%)	1.938	0.164	
CAD	4 (23.5%)	13 (19.4%)	1.317	0.251	
History of stroke or TIA	4 (23.5%)	11 (16.4%)	0.108	0.742	
Smoking	8 (47.1%)	19 (22.4%)	2.056	0.152	
Drinking	6 (35.3%)	19 (28.4%)	0.312	0.576	
TC (mmol/L)	4.25 ± 0.71	4.57 ± 1.08	−1.178	0.242	
TG (mmol/L)	1.69 ± 1.00	1.82 ± 0.86	−0.684	0.496	
LDL (mmol/L)	2.15 ± 0.61	2.42 ± 0.95	−1.128	0.263	
HDL (mmol/L)	1.29 ± 0.512	1.24 ± 0.647	0.122	0.903	
Cr (*μ*mol/L)	62.59 ± 13.72	63.49 ± 13.56	0.258	0.797	
BUN (mmol/L)	5.34 ± 1.42	5.10 ± 1.66	0.568	0.572	
Clopidogrel plus aspirin	3 (17.6%)	24 (35.8%)	2.056	0.152	

BUN: blood urea nitrogen; CAD: coronary artery disease; Cr: creatinine; HDL: high-density lipoprotein; LDL: low-density lipoprotein; MES: microembolic signals; TC: total cholesterol; TG: triglycerides; TIA: transient ischemic attack.

**Table 2:** Plaque ultrasound characteristics within the ipsilateral carotid artery.

	MES+ (*n* = 17)	MES− (*n* = 67)	*T*/*χ*^2^	*P*
IMT	0.960 ± 0.112	0.848 ± 0.152	2.073	0.041
RI	0.768 ± 0.055	0.762 ± 0.054	0.377	0.707
Plaque ulceration	2/17	1/67	3.046	0.110
CAS	9/17	14/67	5.302	0.021
CPL (mm)	23.10 ± 9.18	12.99 ± 8.87	4.167	<0.001
CPT (mm)	2.750 ± 1.135	1.953 ± 750	2.320	0.031
*Plaque echo*				
Echolucent	27/57	51/125	1.426	0.490
Mixed echolucent/echogenic	28/57	65/125		
Echogenic	2/57	9/125		

CAS: carotid artery stenosis; CPL: carotid plaque length; CPT: carotid plaque thickness; IMT: intima-media thickness; MES: microembolic signals; RI: resistance index.

## Data Availability

The data that support this study are available from the responding author upon reasonable request.

## References

[1] Markus H. S., King A., Shipley M. (2010). Asymptomatic embolisation for prediction of stroke in the Asymptomatic Carotid Emboli Study (ACES): a prospective observational study. *The Lancet Neurology*.

[2] Wong K. S. L., Chen C., Fu J. (2010). Clopidogrel plus aspirin versus aspirin alone for reducing embolisation in patients with acute symptomatic cerebral or carotid artery stenosis (CLAIR study): a randomised, open-label, blinded-endpoint trial. *The Lancet Neurology*.

[3] Topakian R., King A., Kwon S. U. (2011). Ultrasonic plaque echolucency and emboli signals predict stroke in asymptomatic carotid stenosis. *Neurology*.

[4] Jiang J., Jiang Y., Feng S. (2013). Microembolic signal monitoring of TOAST-classified cerebral infarction patients. *Molecular Medicine Reports*.

[5] King A., Markus H. S. (2009). Doppler embolic signals in cerebrovascular disease and prediction of stroke risk. *Stroke*.

[6] Goldberg I., Auriel E., Russell D., Korczyn A. D. (2012). Microembolism, silent brain infarcts and dementia. *Journal of the Neurological Sciences*.

[7] Markus H. S., Droste D. W., Kaps M. (2005). Dual antiplatelet therapy with clopidogrel and aspirin in symptomatic carotid stenosis evaluated using doppler embolic signal detection: the Clopidogrel and Aspirin for Reduction of Emboli in Symptomatic Carotid Stenosis (CARESS) trial. *Circulation*.

[8] Spence J. D. (2017). Transcranial Doppler emboli identifies asymptomatic carotid patients at high stroke risk: why this technique should be used more widely. *Angiology*.

[9] Coutinho J. M., Derkatch S., Potvin A. R. J. (2016). Nonstenotic carotid plaque on CT angiography in patients with cryptogenic stroke. *Neurology*.

[10] Ospel J. M., Singh N., Marko M. (2020). Prevalence of ipsilateral nonstenotic carotid plaques on computed tomography angiography in embolic stroke of undetermined source. *Stroke*.

[11] Spence J. D., Hegele R. A. (2004). Non-invasive assessment of atherosclerosis risk. *Current Drug Target -Cardiovascular & Hematological Disorders*.

[12] Elhfnawy A. M., Heuschmann P. U., Pham M., Volkmann J., Fluri F. (2019). Stenosis length and degree interact with the risk of cerebrovascular events related to internal carotid artery stenosis. *Frontiers in Neurology*.

[13] Tang W., Shen X., Li H. (2020). The independent and incremental value of ultrasound carotid plaque length to predict the presence and severity of coronary artery disease: analysis from the carotid plaque length prospective registry. *European Heart Journal-Cardiovascular Imaging*.

[14] Kernan W. N., Ovbiagele B., Black H. R. (2014). Guidelines for the prevention of stroke in patients with stroke and transient ischemic attack: a guideline for healthcare professionals from the American Heart Association/American Stroke Association. *Stroke*.

[15] Brinjikji W., Rabinstein A. A., Lanzino G. (2015). Ultrasound characteristics of symptomatic carotid plaques: a systematic review and meta-analysis. *Cerebrovascular Diseases*.

[16] Touboul P. J., Hennerici M. G., Meairs S. (2007). Mannheim carotid intima-media thickness consensus (2004-2006). An update on behalf of the Advisory Board of the 3rd and 4th Watching the Risk Symposium, 13th and 15th European Stroke Conferences, Mannheim, Germany, 2004, and Brussels, Belgium, 2006. *Cerebrovascular Diseases*.

[17] Muraki M., Mikami T., Yoshimoto T. (2012). New criteria for the sonographic diagnosis of a plaque ulcer in the extracranial carotid artery. *American Journal of Roentgenology*.

[18] Wu X., Zhang H., Liu H., Xing Y., Liu K. (2014). Microembolic signals detected with transcranial Doppler sonography differ between symptomatic and asymptomatic middle cerebral artery stenoses in Northeast China. *PLoS One*.

[19] Müller H. F. G., Viaccoz A., Fisch L. (2014). 18FDG-PET-CT: an imaging biomarker of high-risk carotid plaques. Correlation to symptoms and microembolic signals. *Stroke*.

[20] Cao Y., Cui C., Zhao H. (2019). Plasma osteoprotegerin correlates with stroke severity and the occurrence of microembolic signals in patients with acute ischemic stroke. *Disease Markers*.

[21] Higuchi E., Toi S., Shirai Y. (2020). Prevalence of microembolic signals in embolic stroke of undetermined source and other subtypes of ischemic stroke. *Stroke*.

[22] Zuilen E. V., Van Gijn J., Ackerstaff R. G. A. (1998). The clinical relevance of cerebral microemboli detection by transcranial Doppler ultrasound. *Journal of Neuroimaging*.

[23] Mayor I., Comelli M., Vassileva E., Burkhard P., Sztajzel R. (2003). Microembolic signals and carotid plaque morphology: a study of 71 patients with moderate or high grade carotid stenosis. *Acta Neurologica Scandinavica*.

[24] Liu Y., Hua Y., Liu R. (2018). Ultrasonographical features associated with progression of atherosclerosis in patients with moderate internal carotid artery stenosis. *Translational Stroke Research*.

[25] Kim B. J., Kim J. S. (2014). Ischemic stroke subtype classification: an Asian viewpoint. *Journal of Stroke*.

[26] Zhang H., Li Z., Dai Y., Guo E., Zhang C., Wang Y. (2019). Ischaemic stroke etiological classification system: the agreement analysis of CISS, SPARKLE and TOAST. *Stroke and Vascular Neurology*.

[27] Gao S., Wang Y. J., Xu A. D., Li Y. S., Wang D. Z. (2011). Chinese ischemic stroke subclassification. *Frontiers in Neurology*.

[28] Chao B. H., Yan F., Hua Y. (2021). Stroke prevention and control system in China: CSPPC-Stroke Program. *International Journal of Stroke*.

[29] Arsava E. M., Helenius J., Avery R. (2017). Assessment of the predictive validity of etiologic stroke classification. *JAMA Neurology*.

[30] Bogiatzi C., Wannarong T., McLeod A. I., Heisel M., Hackam D., Spence J. D. (2014). SPARKLE (Subtypes of Ischaemic Stroke Classification System), incorporating measurement of carotid plaque burden: a new validated tool for the classification of ischemic stroke subtypes. *Neuroepidemiology*.

